# Pharmacologic regulation of AMPK in breast cancer affects cytoskeletal properties involved with microtentacle formation and re-attachment

**DOI:** 10.18632/oncotarget.5345

**Published:** 2015-09-23

**Authors:** Kristi R. Chakrabarti, Rebecca A. Whipple, Amanda E. Boggs, Lindsay K. Hessler, Lekhana Bhandary, Michele I. Vitolo, Keyata Thompson, Stuart S. Martin

**Affiliations:** ^1^ University of Maryland School of Medicine, Marlene and Stewart Greenebaum National Cancer Institute Cancer Center, University of Maryland School of Medicine, Baltimore, MD, USA; ^2^ Graduate Program in Molecular Medicine, University of Maryland School of Medicine, Baltimore, MD, USA; ^3^ Department of Pathology and Laboratory Medicine, Perelman School of Medicine, University of Pennsylvania, Philadelphia, PA, USA; ^4^ Department of Physiology, University of Maryland School of Medicine, Baltimore, MD, USA

**Keywords:** AMPK, microtubule stability, microtentacles, breast cancer metastasis, re-attachment

## Abstract

The presence of tumor cells in the circulation is associated with a higher risk of metastasis in patients with breast cancer. Circulating breast tumor cells use tubulin-based structures known as microtentacles (McTNs) to re-attach to endothelial cells and arrest in distant organs. McTN formation is dependent on the opposing cytoskeletal forces of stable microtubules and the actin network. AMP-activated protein kinase (AMPK) is a cellular metabolic regulator that can alter actin and microtubule organization in epithelial cells. We report that AMPK can regulate the cytoskeleton of breast cancer cells in both attached and suspended conditions. We tested the effects of AMPK on microtubule stability and the actin-severing protein, cofilin. AMPK inhibition with compound c increased both microtubule stability and cofilin activation, which also resulted in higher McTN formation and re-attachment. Conversely, AMPK activation with A-769662 decreased microtubule stability and cofilin activation with concurrent decreases in McTN formation and cell re-attachment. This data shows for the first time that AMPK shifts the balance of cytoskeletal forces in suspended breast cancer cells, which affect their ability to form McTNs and re-attach. These results support a model where AMPK activators may be used therapeutically to reduce the metastatic efficiency of breast tumor cells.

## INTRODUCTION

Primary breast cancers shed millions of cells into circulation during the early steps of tumor formation. These single disseminated cells, known as circulating tumor cells (CTCs), can go on to invade distant sites and cause metastatic disease [1]. The presence of CTCs is associated with decreased overall and progression-free survival in patients with metastatic breast cancer [2, 3]. While metastasis remains the leading cause of mortality in patients diagnosed with breast cancer, there are currently no treatments to specifically target the metastatic cascade [4]. A better understanding of CTC biology and specific markers on CTCs are needed for their detection and the development of therapies to treat metastasis.

Activation of the adenosine monophosphate-activated protein kinase (AMPK) pathway has shown great promise in the treatment of breast cancer in pre-clinical models. AMPK is a master regulator of cellular metabolism and works to maintain metabolic homeostasis through its nutrient-sensing capabilities. It is activated by phosphorylation at threonine 172 when the cellular AMP:ATP ratio increases during metabolic stress [5, 6]. Pharmacologic activators of AMPK, such as metformin, phenformin, AICAR, and A-769662, inhibit or delay primary breast tumor growth *in vivo* and induce cell death of breast tumor cells [7–11]. Furthermore, metformin is currently being investigated in a number of clinical trials as a potential adjuvant and/or neoadjuvant therapy for breast cancer patients [12]. A number of non-classical drugs with anti-neoplastic activity have also been shown to activate AMPK as part of their mechanism of action [13]. Therefore, there is currently great interest in developing more selective pharmacological activators of AMPK for clinical use in cancer [14]. Although a great deal of work has been done to study the effects of AMPK on primary tumor formation, its effects on breast cancer metastasis are still largely unknown.

In order to form distant metastases, breast cancer cells must detach from the extracellular matrix (ECM) and enter into the bloodstream or lymphatic system. Once detached, these CTCs undergo a variety of changes, both molecularly and structurally, to adapt to the new microenvironment. After detachment and survival in the circulation, CTCs must re-attach and arrest at a secondary site [15, 16]. Tumor cell re-attachment is a process dependent on stable microtubules [17–21]. Detached breast tumor cells form microtubule-based protrusions, known as microtentacles (McTNs), that aid in CTC aggregation and re-attachment to endothelial cells [19, 22–24]. Therefore, McTNs are critical structures that may be an important therapeutic target to prevent CTC re-attachment.

McTN formation is dependent on the balance of two opposing cytoskeletal forces: the outward force of stabilized microtubules and the inward contractile force of the actin cortex [19]. Currently two post-translational modifications on alpha tubulin, detyrosination and acetylation, play a significant role in McTN formation [22, 25]. Detyrosination removes the C-terminal tyrosine, exposing a glutamic acid residue, and acetylation takes place on the lysine 40 residue of alpha tubulin by alpha tubulin acetyl-transferase (αTAT1/MEC-17) [26, 27]. Both of these modifications are indicators of stabilized microtubules [26–28]. Microtubule stability is associated with greater re-attachment of suspended tumor cells to endothelial monolayers and lung trapping in a murine experimental metastasis model [17, 19, 20, 29]. Increasing glu-tubulin levels, both genetically and pharmacologically, results in greater McTN formation and enhanced suspended cell re-attachment [20, 23, 29, 30]. Elevated acetylated tubulin levels are associated with a higher metastatic phenotype in breast cancer cells and can enhance both McTN formation and re-attachment. In addition, higher levels of acetylated tubulin are enriched in the more aggressive, basal-like subtype of breast cancers and correlate with decreased overall and progression-free survival of breast cancer patients [25]. Conversely, McTNs are antagonized by the actin cytoskeleton. One major regulator of actin that also plays a significant role in McTN formation is the actin-severing protein, cofilin. Cofilin is activated upon dephosphorylation at serine 3, which results in a breakdown of the actin network and increases actin monomers [31]. Activation of cofilin in detached breast epithelial cells promotes McTN formation [24]. There is data to show that AMPK can affect both microtubules and actin in normal epithelial cells [32, 33], but the role of AMPK in regulating the cytoskeleton of breast tumor cells has not yet been investigated.

While the metastatic dissemination of CTCs offers a critical window for cytoskeletal-based therapeutic intervention, microtubule-stabilizing chemotherapies such as taxanes, have cytotoxic side effects and can enhance tumor cell re-attachment [23, 34]. Existing and developing pharmacological AMPK activators that have shown benefit in the primary tumor setting may now also be a potential therapeutic option to decrease the metastatic efficiency of detached breast tumor cells. In this study, we provide a novel role for AMPK in breast cancer. AMPK inhibition with a pharmacologic inhibitor, compound c, significantly increases microtubule stability and cofilin activation, which leads to increased McTN formation and re-attachment in breast cancer cell lines. AMPK activation with A-769662 has an opposing effect, significantly reducing McTNs and tumor cell re-attachment by decreasing cofilin activity and microtubule stability.

## RESULTS

A pharmacological approach was utilized to understand the effects of altering the AMPK pathway on the cytoskeletal properties of breast cancer cells. We chose two different breast cancer cell lines for this study: a non-metastatic, epithelial cell line, MCF-7 [36] and a more invasive, mesenchymal cell line, BT-549 [37], due to their differences in basal AMPK phosphorylation levels and aggressiveness. MCF-7 cells have more epithelial character and thus higher levels of basal AMPK phosphorylation compared to the more mesenchymal BT-549 cells (data not shown). In order to understand how AMPK-directed drugs would affect the cytoskeleton of these two different cell types, we used the small molecules compound c and A-769662. Compound c (dorsomorphin dihydrochloride) can potently block AMPK activation [38, 39]. A-769662 can allosterically activate AMPK and also inhibit the dephosphorylation of AMPK on threonine 172, making it more specific for AMPK [40, 41]. Previous studies using these drugs in breast cancer have focused solely on their effects on cell growth and death [7, 8, 42]. In this study, we were interested in understanding the effects of these drugs on the cytoskeleton at earlier time points.

### AMPK inhibition increases cofilin activation and microtubule stability

**Figure 1:**
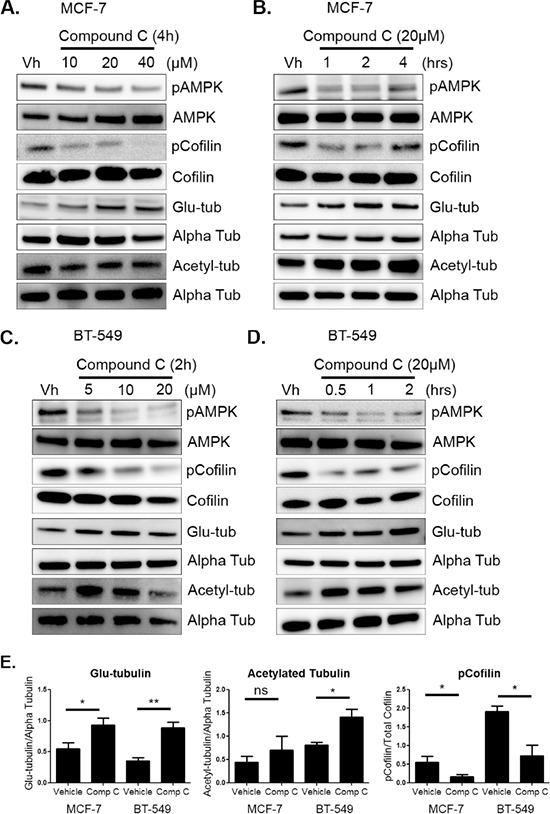
AMPK inhibition increases cofilin activation and microtubule stability **A & B.** MCF-7 cells were treated with 10–40 μM of compound c in serum-free media for 1–4 hours. Protein was harvested and Western blot analysis was done to determine levels of pAMPK at threonine 172, pCofilin at serine 3, detyrosinated alpha tubulin (glu-tub), and acetylated alpha tubulin (acetyl-tub). **C & D.** BT-549 cells were treated with 5–20 μM compound c in serum-free media for 30 mins to 2 hours. Protein was harvested and Western blot analysis was done to determine levels of markers listed above. **E.** Densitometry was done to quantify levels of pCofilin, glu-tubulin, and acetylated tubulin from at least three independent experiments (mean +/− SEM) in MCF-7 and BT-549 cells treated with 20 μM compound c for 4 and 2 hours, respectively. *p < 0.05. **p < 0.005. *ns* = not significant.

MCF-7 and BT-549 cells were first treated, under attached conditions, with varying doses of compound c to find the optimum dose for AMPK inhibition (Figure 1A and 1C). A dose of 20 μM was chosen based on maximum pAMPK inhibition at threonine 172. MCF-7 and BT-549 cells were treated with 20 μM compound c for varying times (Figure 1B and 1D) to determine the optimum time course of treatment was less than 4 hours. Drug treatment did not induce cytotoxicity at these early time points (Supplementary Figure S1A). Using an assay that is independent of cellular metabolism [35], we found that compound c had comparable effects on cell growth in MCF-7 and BT-549 cells (Supplementary Figure S2A and S2B) as previously established [39].

Compound c treatment also resulted in changes in cofilin activation and microtubule stability at these early time points. With AMPK inhibition, there was a corresponding increase in cofilin activation in both cell lines, as indicated by a dose-dependent decrease in cofilin phosphorylation (Figure 1A and 1C). Significant cofilin activation was achieved by 2 hours in MCF-7 and 2 BT-549 cells (Supplementary Figure S3A and 3B). In addition to changes in the actin cortex, microtubule stability was also enhanced in both cell lines. There was an increase in glu-tubulin levels at 4 hours in MCF-7 cells and BT-549 cells were slightly more sensitive with an increase appearing by 1 hour (Figure 1B and 1D). Densitometry revealed a significant increase in glu-tubulin in MCF-7 cells at 4 hours and by 2 hours in BT-549 cells (Figure 1E and Supplementary Figure S3A and S3B). BT-549 cells also significantly increased tubulin acetylation by 2 hours while MCF-7 cells had a modest increase that was not significant (Figure 1E).

**Figure 2:**
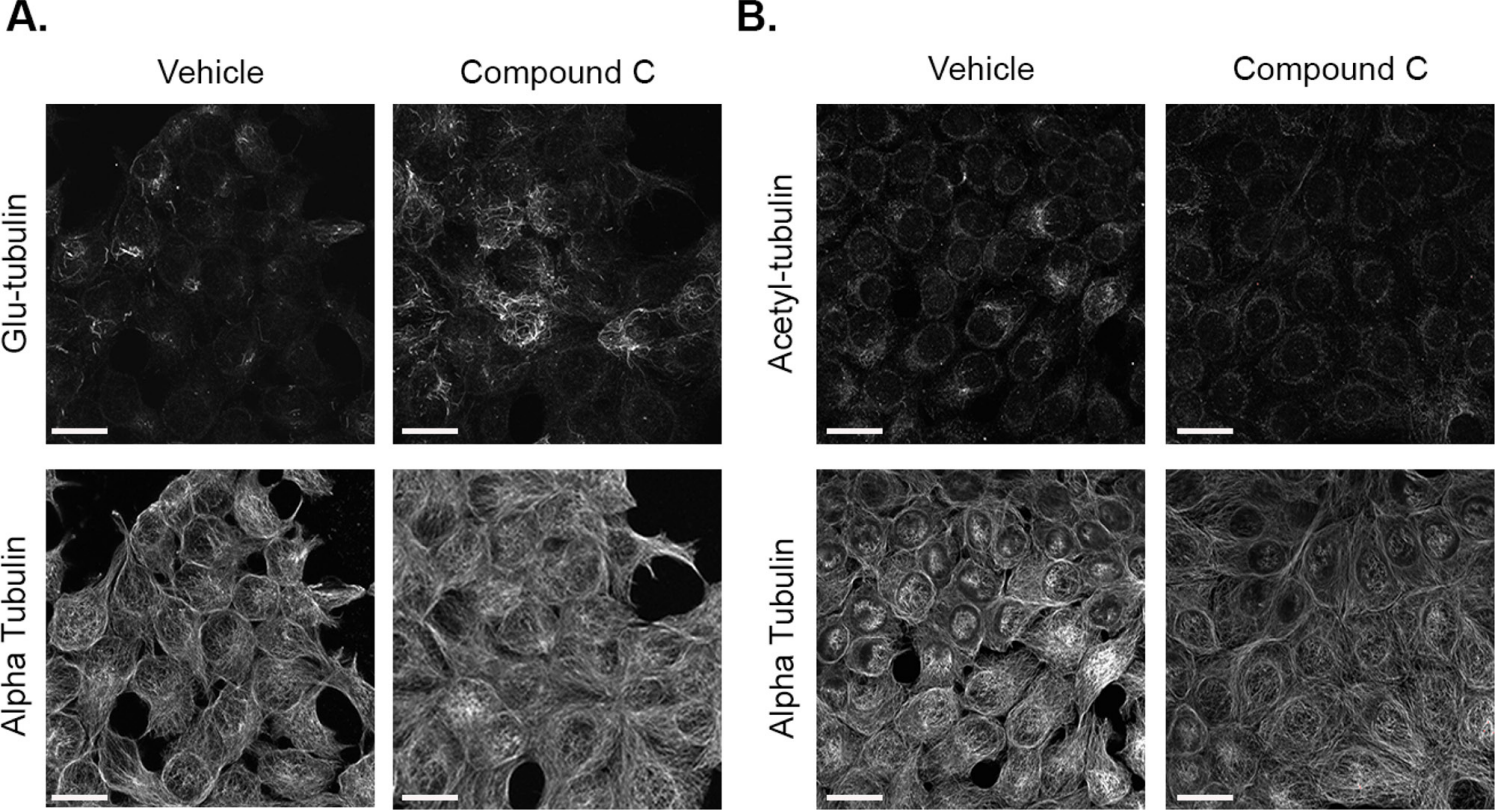
Compound c increases glu-tubulin levels in MCF-7 cells MCF-7 cells treated with 20 μM compound c for 4 hours in serum-free media were fixed and stained with **A.** glu-tubulin and alpha tubulin. **B.** Acetylated tubulin and alpha tubulin. All images were taken at 60× magnification and are displayed as maximum z projections. Scale bar corresponds to 20 μm.

In order to visualize the effects of compound c on microtubules, immunofluorescence was performed on both cell lines to detect changes in detyrosinated and acetylated alpha tubulin. MCF-7 and BT-549 cells were treated with 20 μM compound c for 4 hours and 2 hours, respectively. Compound c slightly increased detyrosination of alpha-tubulin (Figure 2A), but no appreciable change was seen in acetylated tubulin in MCF-7 cells (Figure 2B). BT-549 cells had higher basal levels of glu-tubulin, but compound c treatment resulted in more filamentous glu-tubulin (Supplementary Figure S4A) and also increased acetylated tubulin (Supplementary Figure S4B). Compound c treatment did not alter the alpha tubulin network in either cell line. Therefore, 2 to 4 hours of AMPK inhibition increased microtubule stability in both MCF-7 and BT-549 breast cancer cells.

### Compound c increases activated cofilin and tubulin acetylation in suspended cells

Once the effects of compound c on attached breast tumor cells were established, MCF-7 and BT-549 cells were pre-treated with compound c as described above and then suspended in drug, as an *in vitro* model of detached tumor cells. Based on the cytoskeletal changes seen in the attached cells, MCF-7 cells were pretreated for 4 hours and BT-549 cells were pretreated for 2 hours with 20 μM compound c. Cells were then harvested at varying time points to analyze the effects of AMPK inhibition on microtubule stability and the actin network under suspended conditions.

**Figure 3:**
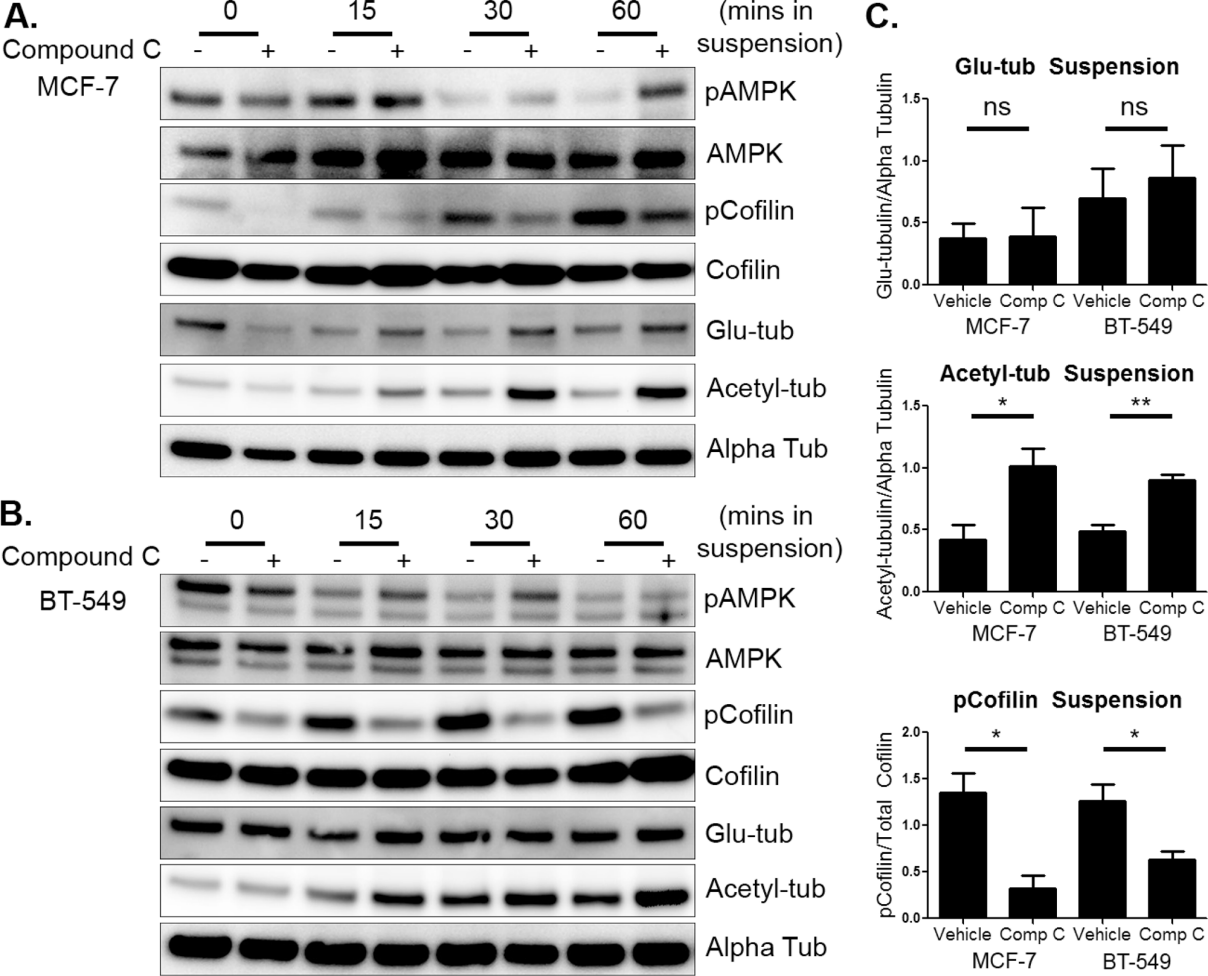
Compound c increases activated cofilin and tubulin acetylation in suspended cells **A.** MCF-7 cells were treated with 20 μM compound c for 4 hours in serum-free media and then suspended. Cells were harvested immediately after suspension (0 min) and then harvested at 15, 30, and 60 minutes. Western blot analysis was done to determine levels of pAMPK, pCofilin, glu-tubulin, and acetylated tubulin. **B.** BT-549 cells were treated with 20 μM compound c for 2 hours in serum-free media and suspended. Cells were harvested and Western blot analysis was done as above. **C.** Densitometry was done at 60 minutes of suspension of three independent experiments (mean +/− SEM) to quantify levels of pCofilin, glu-tubulin, and acetylated tubulin. *p < 0.05 **p < 0.005. *ns* = not significant.

Even though AMPK phosphorylation was lost over time in suspension and the cells were able to rapidly recover from drug treatment, compound c had a sustained effect on the cytoskeleton in suspension. The combination of compound c pretreatment and the initial inhibition of the AMPK pathway at time 0 in suspension were sufficient to maintain cofilin activation (Figure 3A and 3B). Acetylated tubulin levels increased in MCF-7 and BT-549 cells by 15 minutes in suspension, with the biggest difference appearing around 60 minutes. Glu-tubulin levels did not change in either cell line in suspension with treatment (Figure 3A and 3B). Densitometry at 60 minutes of suspension indicated both cell lines significantly increased cofilin activation and acetylated tubulin (Figure 3C). Compound c treatment and suspension also did not induce cytotoxicity (Supplementary Figure S1C). Therefore, compound c increased microtubule stability and cofilin activity in detached human breast tumor cells.

### AMPK inhibition increases microtentacles and tumor cell re-attachment

**Figure 4:**
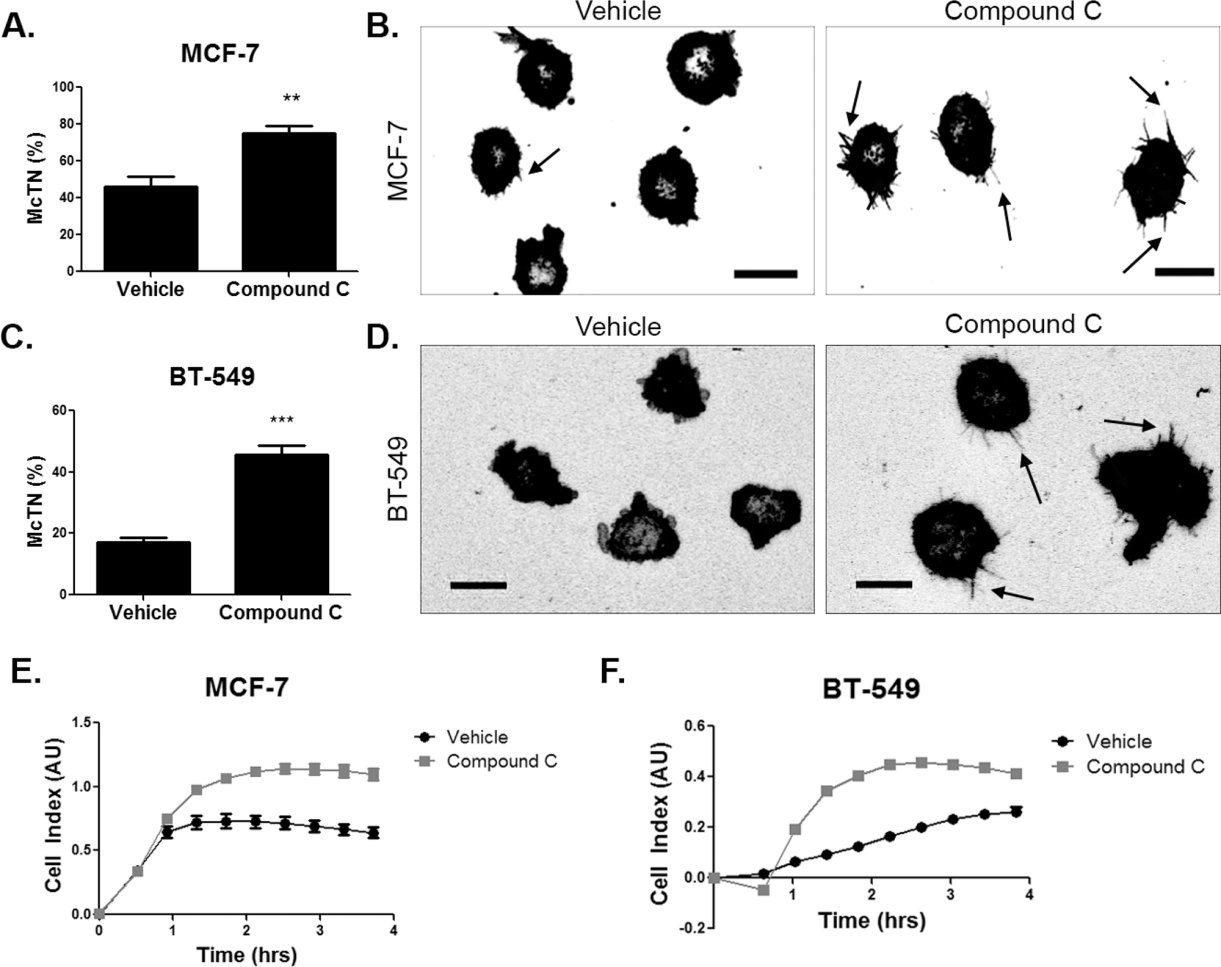
AMPK inhibition increases microtentacles and tumor cell re-attachment **A.** MCF-7 cells were suspended after treatment with 20 μM compound c for 4 hours in serum-free media and stained with CellMask Orange cell membrane dye. Blinded quantification of microtentacle frequency from three independent experiments (mean +/− SEM) with 100 cells counted for each was averaged. **p < 0.001. **B.** Representative images show microtentacles (arrow) on vehicle control and compound c treated MCF-7 cells. Scale bar corresponds to 20 μm. **C.** BT-549 cells were suspended after treatment with 20 μM compound c for 4 hours in serum-free media and stained with CellMask Orange cell membrane dye. Blinded quantification of microtentacle frequency from three independent experiments (mean +/− SEM) with 100 cells counted for each were averaged. ***p < 0.0001. **D.** Representative images show microtentacles (arrow) on vehicle control and compound c treated BT-549 cells. Scale bar corresponds to 20 μm. **E & F.** Re-attachment of suspended MCF-7 and BT-549 cells treated with 20 μM compound c for 4 hours and 2 hours, respectively, in serum-free media using the real time xCELLigence analyzer.

We next wanted to determine if the alterations in the cytoskeleton upon compound c treatment resulted in changes in McTN formation and tumor cell re-attachment. MCF-7 cells were pretreated for 4 hours and BT-549 cells were pretreated for 2 hours with 20 μM compound c and McTN counts were conducted after 15–30 minutes in suspension. Blinded McTN quantification found that MCF-7 and BT-549 cells treated with compound c significantly increased McTN frequency, as compared to cells treated with vehicle control (Figure 4A and 4C). Representative images show the increase in McTNs in compound c treated cells (Figure 4B and 4D).

In order to characterize the functional role of the McTNs, we measured the re-attachment of suspended breast tumor cells to model the early steps of CTC arrest in distant tissues [20]. The increase in McTNs upon compound c treatment corresponded to greater tumor cell re-attachment (Figure 4E and 4F). Re-attachment was increased in both cell lines with compound c treatment by 1–2 hours of suspension. Inhibiting AMPK in suspended breast tumor cells resulted in higher McTN formation and re-attachment, which corresponded with higher microtubule stability and cofilin activation.

### AMPK activation decreases cofilin activation and tubulin acetylation in attached cells

In complementary experiments, we sought to understand if AMPK activation could reverse the effects on the cytoskeleton and McTNs seen with AMPK inhibition. AMPK activation has been previously shown to have therapeutic value in breast cancer cell growth in pre-clinical models [43]. At the dose and time point we used, there was no cytotoxicity (Supplementary Figure S1B), but we did see similar results on cell growth at 48 hours with A-769662 treatment as previously reported (Supplementary Figure S2C) [10].

**Figure 5:**
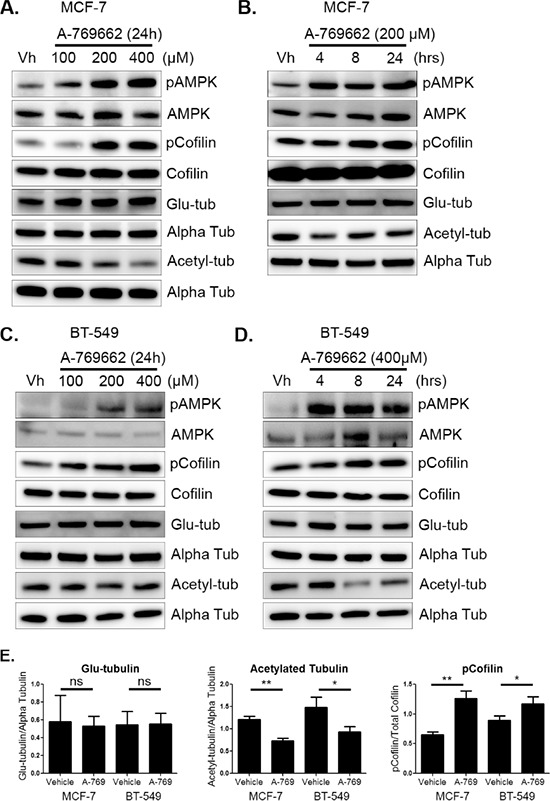
AMPK activation decreases cofilin activation and acetylated tubulin **A & B.** MCF-7 cells were treated with 100–400 μM of A-769662 for 4–24 hours. Protein was harvested and Western blot analysis was done to determine levels of pAMPK at threonine 172, pCofilin at serine 3, detyrosinated alpha tubulin (glu-tub), and acetylated alpha tubulin (acetyl-tub). **C & D.** BT-549 cells were treated with 100–400 μM A-769662 for 4–24 hours. Protein was harvested and Western blot analysis was done to determine levels of markers listed above. **E.** Densitometry was done to quantify levels of pCofilin, glu-tubulin, and acetylated tubulin from three independent experiments (mean +/− SEM) in MCF-7 and BT-549 cells treated with 200 μM and 400 μM A-769662, respectively, for 24 hours. *p < 0.05. **p < 0.005. *ns* = not significant.

Attached MCF-7 cells were treated with varying doses of A-769662 for 24 hours. There was robust activation of AMPK in MCF-7 cells at 200 μM (Figure 5A). AMPK activation also resulted in a significant increase in cofilin phosphorylation with 200 μM A-769662 treatment by 8 hours (Figure 5B and Supplementary Figure S3C). There was no change in glu-tubulin levels, but acetylated tubulin decreased with AMPK activation in a time-dependent manner (Figures 5A, 5B, and Supplementary Figure S3C). BT-549 cells activated AMPK with 400 μM A-769662 at 4 hours, which was maintained for 24 hours (Figure 5C and 5D). Similar to MCF-7 cells, AMPK activation resulted in increased cofilin phosphorylation with 400 μM A-769662 by 8 hours and was sustained for 24 hours. Tubulin acetylation also decreased upon AMPK activation with 400 μM treatment by 8 hours (Figure 5D and Supplementary Figure S3D). Densitometry at 24 hours showed a significant decrease in cofilin activation and acetylated tubulin in MCF-7 cells and BT-549 cells with no change in glu-tubulin (Figure 5E).

**Figure 6:**
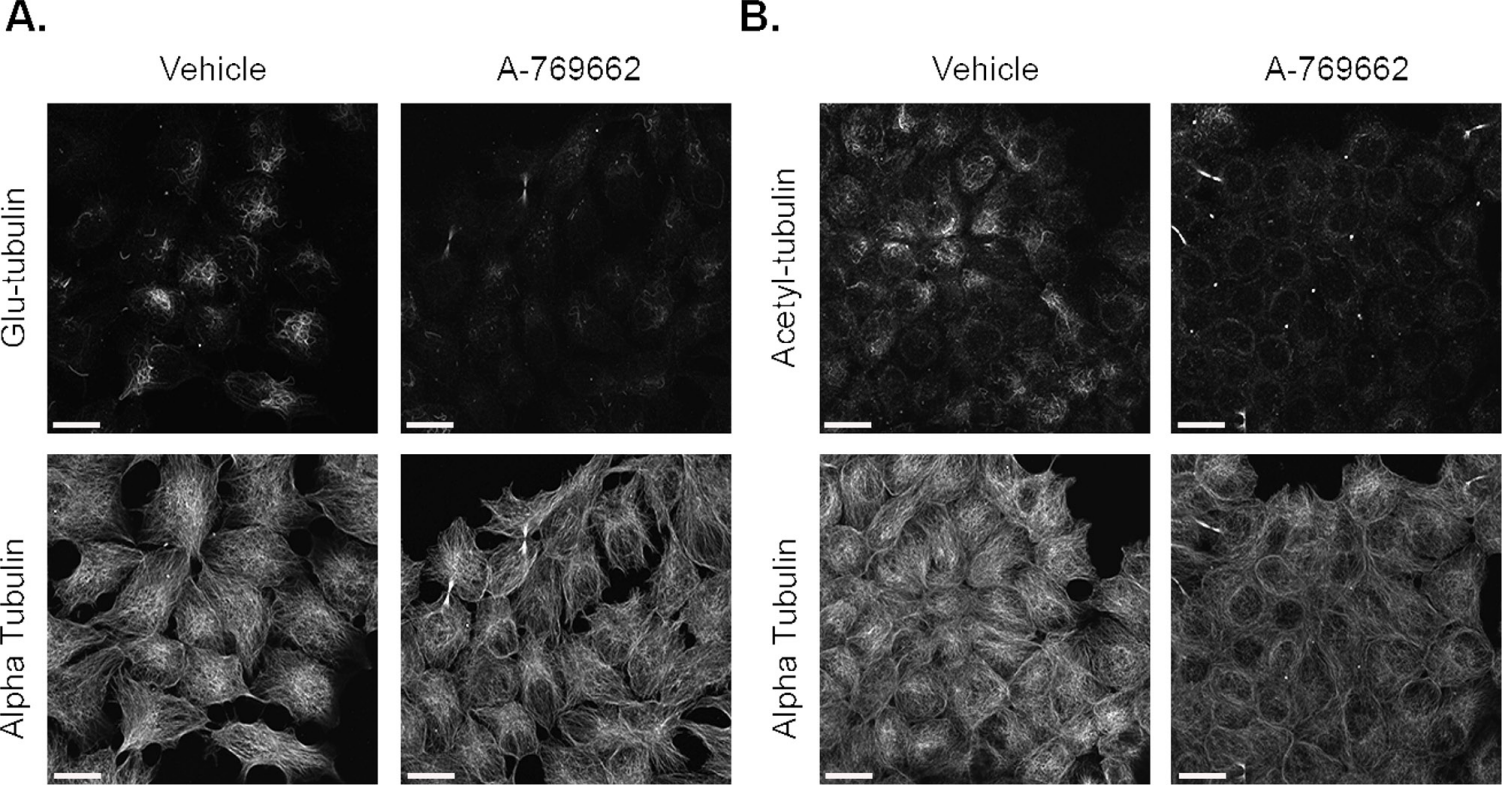
A-769662 does not change glu-tubulin or acetylated tubulin in MCF-7 cells MCF-7 cells treated with 2000 μM A-769662 for 24 hours were fixed and stained with **A.** glu-tubulin and alpha tubulin. **B.** Acetylated tubulin and alpha tubulin. All images were taken at 60× magnification and are displayed as maximum z projections. Scale bar corresponds to 20 μm.

In addition to overall protein levels, changes in microtubule stability in MCF-7 and BT-549 cells with A-769662 treatment were visualized with immunofluorescence. There was no appreciable change in glu-tubulin levels or organization in MCF-7 cells, which mimicked the Western blot results (Figure 6A). MCF-7 cells slightly decreased overall acetylation with 200 μM A-769662 for 24 hours (Figure 6B). BT-549 cells also showed no difference in glu-tubulin (Supplementary Figure S5A), but there was a more marked decrease in tubulin acetylation with 400 μM A-769662 treatment for 24 hours (Supplementary Figure S5B). Alpha tubulin did not change with treatment as a control.

### AMPK activation decreases cofilin activation in MCF-7 cells and microtubule stability in BT-549 cells in suspension

**Figure 7:**
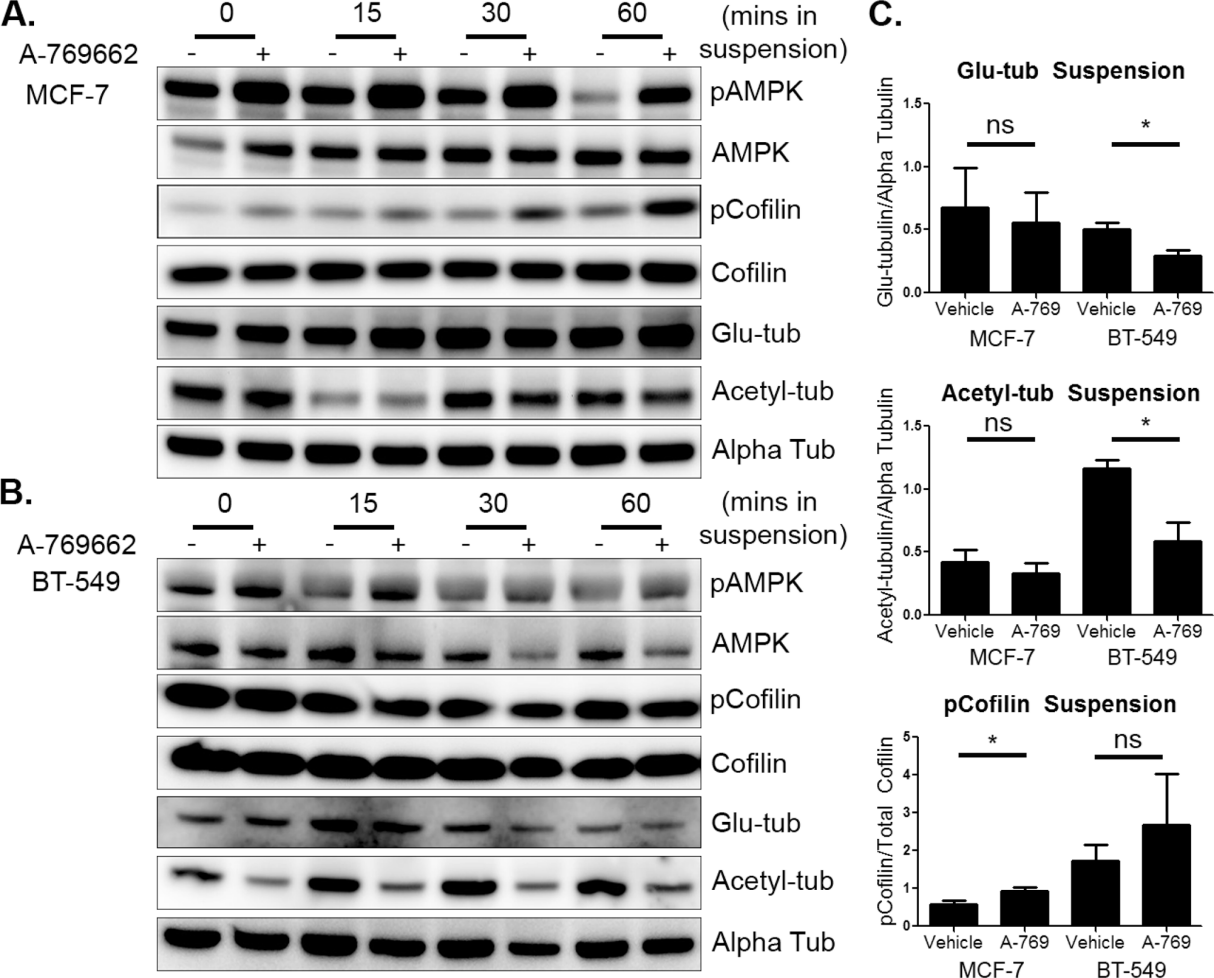
AMPK activation in suspended cells has differential effects on microtubule stability and cofilin activation **A.** MCF-7 cells were treated with 200 μM A-769662 for 24 hours and then suspended. Cells were harvested immediately after suspension (0 min) and then harvested at 15, 30, and 60 minutes. Western blot analysis was done to determine levels of pAMPK, pCofilin, glu-tubulin, and acetylated tubulin. **B.** BT-549 cells were treated with 400 μM A-769662 for 24 hours and suspended. Cells were harvested and Western blot analysis was done as above. **C.** Densitometry was done at 60 minutes of suspension of three independent experiments (mean +/− SEM) to quantify levels of pCofilin, glu-tubulin, and acetylated tubulin. *p < 0.05 **p < 0.005. *ns* = not significant.

Based on the changes detected in attached cells, MCF-7 and BT-549 cells were pretreated with 200 μM and 400 μM A-769662, respectively, for 24 hours and then suspended to test the cytoskeletal effects of A-769662 on detached breast tumor cells. Both cell lines activated AMPK through increased phosphorylation even though basal phosphorylation levels did decline over time as seen previously. Similar to the attached cells, MCF-7 cells decreased cofilin activation with A-769662 treatment starting at 15 minutes with the biggest changes seen at 60 minutes (Figure 7A and 7C). There were only subtle changes in microtubule stability seen in suspended MCF-7 cells. BT-549 cells, on the other hand, had little to no change in cofilin activation in suspension, but did change microtubule stability (Figure 7B). Alpha tubulin acetylation decreased dramatically at every time point with A-769662 treatment. Glu-tubulin levels decreased, starting around 15–30 minutes of suspension, but did not have as robust of a change compared to tubulin acetylation. Densitometry revealed that MCF-7 cells significantly decreased cofilin activation after 60 minutes of suspension, but did not alter microtubule stability. The opposite effect was seen in BT-549 cells where there was a significant decrease in microtubule stability at 60 minutes of suspension, but cofilin did not change (Figure 7C). Drug treatment and suspension did not induce cytotoxicity (Supplementary Figure S1D).

### AMPK activation decreases microtentacles and tumor cell re-attachment

**Figure 8:**
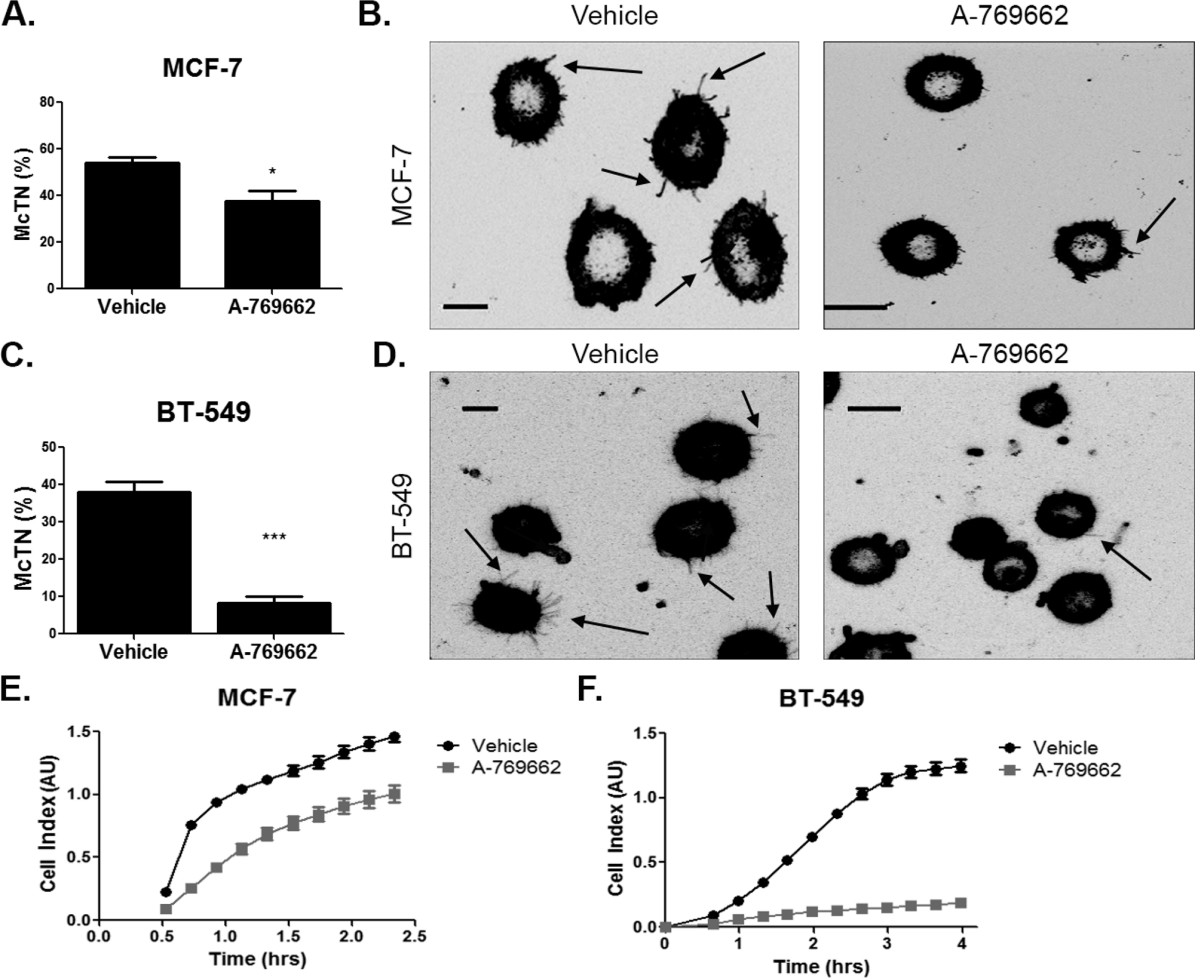
AMPK activation decreases microtentacles and tumor cell re-attachment **A.** MCF-7 cells were suspended after treatment with 200 μM A-769662 for 24 hours and stained with CellMask Orange cell membrane dye. Blinded quantification of microtentacle frequency from three independent experiments (mean +/− SEM) with 100 cells counted for each was averaged. *p < 0.05. **B.** Representative images show microtentacles (arrow) on vehicle control and A-769662 treated cells. **C.** BT-549 cells were suspended after treatment with 400 μM A-769662 for 24 hours and stained with CellMask Orange cell membrane dye. Blinded quantification of microtentacle frequency from three independent experiments (mean +/− SEM) with 100 cells counted for each was averaged. ***p < 0.0005. **D.** Representative images show microtentacles (arrow) on vehicle control and A-769662 treated cells. **E & F.** Re-attachment of suspended MCF-7 and BT-549 cells treated with 200 μM and 400 μM A-769662, respectively, for 24 hours using the real time xCELLigence analyzer.

Since AMPK activation decreased microtubule stability in BT-549 cells and cofilin activation in MCF-7 cells in suspension, we measured its effect on McTN formation and re-attachment. Both MCF-7 and BT-549 cells significantly decreased McTN frequency with 200 μM and 400 μM A-769662 treatment, respectively, for 24 hours (Figure 8A and 8C). There was a more robust decrease in McTN formation in BT-549 cells compared to MCF-7 cells. Representative images show a decrease in McTNs in a population of treated cells (Figure 8B and 8D) compared to vehicle control. The decrease in McTNs also corresponded with a decrease in cell re-attachment in MCF-7 and BT-549 cells (Figure 8E and 8F). MCF-7 cells decreased re-attachment starting at about 30 minutes. A-769662 was also able to better impede BT-549 cell re-attachment starting at slightly over 1 hour of suspension.

## DISCUSSION

This study investigates the role of the cellular metabolic regulator, AMPK, on the cytoskeletal properties of human breast tumor cells. The AMPK pathway has long been known to have numerous downstream signaling functions affecting cellular metabolism, protein synthesis, cell growth, and autophagy to maintain cellular metabolic homeostasis [43]. In this study, we show that AMPK regulates the cytoskeleton by altering both microtubule stability and the actin network in attached and detached breast cancer cells. This suggests a novel role for AMPK in breast cancer.

Here we report that the AMPK pathway can alter post-translational modifications on tubulin in MCF-7 and BT-549 human breast tumor cells. AMPK inhibition with compound c significantly increased both glu-tubulin and acetylated tubulin in BT-549 cells and increased only glu-tubulin in MCF-7 cells. While there are cell type differences, both modifications indicate an increase in microtubule stability with compound c treatment. This supports a previous study where AMPK inhibition with compound c increased glu-tubulin levels by decreasing the phosphorylation of a microtubule plus-end binding protein, clip-170, in normal kidney epithelial (Vero) cells [33]. Increased expression of glu-tubulin and acetylated tubulin are associated with poor prognosis in breast cancer [25, 44] and acetylated tubulin acts as a prognostic marker in head and neck squamous cell carcinoma [45]. In addition, these markers of stable microtubules are found in the invasive front of tumors, aid in epithelial to mesenchymal transition (EMT), and enhance tumor cell migration [25, 29]. Therefore, inhibiting AMPK with compound c may raise the metastatic potential of breast tumor cells and may not be a beneficial therapeutic strategy in some settings.

Conversely, we show that AMPK activation with A-769662 decreased a post-translational modification on tubulin, serving as an indicator that AMPK activation reduces microtubule stability. When attached, treatment of MCF-7 and BT-549 cells with A-769662 decreased tubulin acetylation but did not change glu-tubulin. There is currently very little known about the association of AMPK with microtubule acetylation in breast cancer. Previous work in HeLa cells have found that AMPK activation in response to stress causes MEC-17 phosphorylation, which actually hyperacetylates microtubules [46]. However, it is not known if MEC-17 is a direct substrate of AMPK. Further, MEC-17 can be regulated by a variety of mechanisms, including auto-acetylation, that can alter microtubule acetylation levels [47]. It has been previously shown that treatment of Vero cells with the AMPK activator, AICAR, did not change glu-tubulin levels, but in cardiomyocytes AICAR treatment decreased glu-tubulin [33, 48]. However, neither of these studies measured acetylation. Based on our data and the current literature, it is clear that AMPK is able to regulate microtubule stability as a response to cell stress. Further research is required to understand the mechanism by which AMPK can alter microtubule stability using genetic mutations in AMPK and other tubulin regulatory proteins such as MEC-17.

McTNs and tumor re-attachment are regulated not only by the outward force of stabilized microtubules, but also the inward contractile force of the actin cortex [17, 19]. Here we identify, for the first time in breast cancer, that AMPK can modulate cofilin phosphorylation. Previous work has demonstrated that AMPK activation with A-769662 can remodel the actin cytoskeleton in polarized epithelial cells by phosphorylating key actin regulatory proteins, including cofilin [32]. However, a direct relationship between AMPK and cofilin phosphorylation has not been established. AMPK activation has also been shown to reduce cell motility by inhibiting focal adhesion kinase in tumor cells and can actually suppress metastasis in animals [49]. Here we find that AMPK inhibition decreased cofilin phosphorylation in both MCF-7 and BT-549 cell lines. Activation of AMPK with A-769662 increased cofilin phosphorylation in attached breast cancer cells, which supports work done in Madin-Darby canine kidney cells where A-769662 treatment also raised cofilin phosphorylation [32]. The cofilin pathway is a central player in the generation of actin-based structures such as lamellipodia, invadopodia, and filopodia, which are essential for cell migration and invasion [50–53]. Further work is required to determine how AMPK specifically regulates cofilin phosphorylation to improve targeting of this pathway with the goal of reducing the invasive and metastatic phenotype of tumor cells.

Our results show for the first time that AMPK also plays a role in regulating the cytoskeleton of detached breast tumor cells, which affects their McTN formation and re-attachment ability. AMPK can play an important role in regulating the balance of both actin and microtubules in suspended breast tumor cells. We chose to examine MCF-7 and BT-549 cells because of their differences in AMPK levels and invasiveness so it is predicted that their cytoskeleton would also have distinctive responses to AMPK-directed drugs. Though the two cell lines mostly responded similarly to drug treatments, they did show slight differences in sensitivity to each drug. AMPK activation with A-769662 in suspended conditions caused the most cell-line specific changes as MCF-7 cells decreased cofilin activation, but BT-549 cells responded to AMPK activation by decreasing microtubule stability. Ultimately AMPK activation reduced McTN frequency in both cell lines and diminished their ability to re-attach. It has previously been established that increased cofilin activity leads to enhanced McTN formation and re-attachment, supporting our current data [24]. The re-attachment process is a key early step for CTC retention at a distant site [16]. Therefore, our data shows that AMPK activation may lower the metastatic efficiency of circulating breast tumor cells through its regulation of the cytoskeleton.

AMPK activation has already shown great promise in treating primary breast cancer in pre-clinical models [14]. However, debate still remains in the field on the therapeutic benefit of AMPK in cancer [54]. While AMPK's adaptive response to cell stress in cancer may provide a survival benefit to tumor cells and allow them to withstand states of decreased nutrient availability [55–57], many studies have suggested that the LKB1-AMPK axis has tumor suppressor properties [11, 43]. LKB1 is the upstream kinase that phosphorylates AMPK and is a tumor suppressor that is mutated in Peutz-Jeghers syndrome (PJS) [58]. Patients with PJS have a 54% increased risk in developing breast cancer [59] and LKB1 is mutated in 30% of sporadic breast cancer [42]. AMPK is also known to inhibit cell growth by regulating p53 and other cell cycle proteins [9, 42, 54, 59]. In addition, AMPK activators such metformin and A-769662 inhibit tumor growth in xenograft models and induce growth arrest *in vitro* [7, 11, 42].

In particular, ECM-detached epithelial cells are known to be metabolically compromised because of their inability to take up glucose and produce ATP at the same rate [60]. ECM-detached immortalized normal breast epithelial cells, MCF-10A, activate AMPK upon detachment [61]. While normal epithelial cells undergo programmed cell death in these conditions [60, 62], we and others have found that suspended breast tumor cells also activate AMPK when detached [61] and have enhanced survival in suspension compared to MCF-10A cells [61, 63]. Unique to this study, we focused on early time points after tumor cell suspension to model the particular step of re-attachment in the metastatic cascade. Most other work has focused on 24 to 48 hours of suspension of breast epithelial or tumor cells to assay survival. However, CTCs are able to actively adhere to the vasculature within a much shorter timeframe than 24 hours of dissemination into the circulation [16]. During this time, adhesion of tumor cells with other cells can contribute to survival in the circulation [20]. McTNs promote heterotypic and homotypic cell-cell aggregation which may protect CTCs while they are in transit [20, 23, 24, 64]. Permanent arrest at a distant organ requires adhesive interactions of tumor cells with the endothelium, which can also be facilitated by McTNs on the surface of tumor cells [16]. By showing that AMPK activation significantly decreases McTN formation and re-attachment of breast tumor cells, we report a novel role for AMPK during the early stage of metastatic dissemination.

Though an abundance of work exists on understanding AMPK signaling in primary tumor growth and progression, the role of AMPK in metastasis is still unclear. The main function of AMPK activation in a cell is to revert to a state of increased catabolism and decreased anabolism to return the cell to homeostasis [5, 6]. Therefore, pathways such as protein synthesis are mostly turned off. This study offers new insight on AMPK's role in breast tumor cell biology through regulation of the cytoskeleton. Elevating and/or sustaining AMPK activation by treating cells with an AMPK activator could have global consequences for the cell's cytoskeleton that may reduce McTN formation to conserve energy. We show for the first time in breast cancer that AMPK activation can destabilize microtubules while strengthening the actin network by inhibiting cofilin. These molecular changes result in a decrease in the formation of McTNs in suspended breast tumor cells that attenuate their ability to re-attach. In this manner, we can take advantage of current AMPK activators that are already available or in clinical development to increase the survival of patients with breast cancer by decreasing the metastatic potential of detached breast tumor cells.

## MATERIALS AND METHODS

### Cell culture and reagents

BT-549 and MCF-7 human breast cancer cell lines were obtained from ATCC and cultured according to their instructions. MCF-7 cells were cultured in Dulbecco's Modified Eagle Media (Corning) supplemented with 10% fetal bovine serum (FBS) and 1% penicillin-streptomycin solution (P/S). BT-549 cells were cultured in RPMI-1640 (Corning) supplemented with 1 μg/ml insulin, 10% FBS, and 1% P/S. All cells were maintained at 37°C with 5% CO_2_. The AMPK inhibitor, compound c, was obtained from EMD Millipore and the AMPK activator, A-769662, was obtained from Tocris Bioscience.

### Western blot analysis

MCF-7 and BT-549 cells were treated with 20 μM compound c in serum-free media for 1 hr, 2 hrs, and 4 hrs. MCF-7 and BT-549 cells were also treated at different concentrations of compound c and harvested at 4 and 2 hours, respectively. MCF-7 and BT-549 cells were treated in full-serum media with 200 μM and 400 μM of A-769662, respectively, for 4 hrs, 8 hrs, and 24 hrs. MCF-7 and BT-549 cells were treated at different concentrations of A-769662 and harvested after 24 hours. Attached cells were harvested using ice cold RIPA buffer (EMD Millipore) with 1 mM PMSF, phosphatase inhibitor cocktail (EMD Millipore), and protease inhibitor cocktail (Sigma-Aldrich). Cells were detached using a cell lifter (Corning) or trypsin and transferred to a low-attach plate (Corning) and harvested after 0, 15, 30, and 60 minutes in suspension. Cells were collected and spun at 2,000 rpm for 5 minutes. The pellet was washed with PBS and then resuspended in RIPA buffer. Protein concentration was determined by DC protein assay (Bio-Rad). Equal amounts of lysate were heated with 4x SDS sample buffer containing 0.1 mM DTT. Protein samples were loaded on a 4–12% NuPAGE Bis-Tris gel (Life Technologies). Gels were transferred to a polyvinylidene difluoride membrane (Bio-Rad). Membranes were blocked in 5% (w/v) milk in tris-buffered saline pH 7.4 with 0.5% tween (TBS-T) or 5% (w/v) bovine serum albumin (BSA) in TBS-T for phospho-specific antibodies for 1 hour at room temperature (RT). All antibodies were used at a 1:1000 dilution in 2.5% (w/v) milk in TBS-T or 5% BSA in TBS-T for phospho-specific antibodies and incubated overnight at 4°C. Primary antibodies: Detyrosinated alpha tubulin (Abcam), PARP-1 (Santa Cruz), alpha-tubulin (Sigma-Aldrich), and the following from Cell Signaling Technologies: Acetylated tubulin lysine 40, pAMPK T172, AMPK, pCofilin S3, and Cofilin. Secondary anti-mouse and anti-rabbit IgG horse radish peroxidase was used at 1:10,000 and 1:5,000 dilution, respectively (Jackson ImmunoResearch Laboratories). Signal was detected using Amersham ECL prime western blotting detection reagent (GE Healthcare Life Sciences). Densitometry was performed using ImageJ (NIH) and statistical significance was determined using student's *t*-test comparing treated and vehicle control groups from at least three independent experiments

### Immunofluorescence

MCF-7 and BT-549 cells were cultured on glass coverslips and treated with 20 μM compound c in serum-free media for 4 and 2 hours, respectively. MCF-7 and BT-549 cells were cultured on glass coverslips and treated with 200 μM and 400 μM A-769662, respectively, for 24 hours. Cells were fixed for 10 minutes in ice cold methanol. Cells were permeabilized with 0.25% Triton X-100 for 10 minutes and blocked in 5% BSA/0.5% NP-40 solution for 1 hour at RT. Primary antibody for detyrosinated tubulin, acetylated tubulin, and alpha tubulin was diluted (1:500) in 2.5% BSA/0.5% NP-40 and incubated overnight at 4°C. Secondary anti-mouse Alexa594 and anti-rabbit Alexa488 antibodies (1:1,000; Life Technologies) were diluted in PBS and incubated for 1 hour at RT. Coverslips were mounted on glass slides using Fluoromount-G (Southern Biotech). Images were taken using Olympus FV1000 confocal microscope at 60x magnification under the same exposure settings for corresponding vehicle and drug treatments.

### McTN scoring

MCF-7 and BT-549 cells were treated with 20 μM compound c in serum-free media for 4 and 2 hours, respectively. MCF-7 and BT-549 cells were treated with 200 μM and 400 μM A-769662, respectively, for 24 hours. Cells were stained with CellMask Orange (1:5,000; Life Technologies) in PBS for 5 minutes at 37°C. Cells were detached with 0.25% trypsin-EDTA (Corning) and resuspended in DMEM without phenol-red or serum with corresponding dose of drug and transferred to a low-attach plate (Corning). McTNs were scored blindly in a population of 100 cells/well as previously described [22]. McTNs were imaged after CellMask Orange staining using the Nikon A1R confocal microscope and a maximum z-projection was generated. Images were then inverted using ImageJ. Statistical significance was determined using student's *t*-test comparing McTN frequency in treated and vehicle control groups from three independent experiments.

### Re-attachment assay

MCF-7 and BT-549 cells were treated with 20 μM compound c for 4 and 2 hours, respectively in serum-free media. MCF-7 and BT-549 cells were cultured and treated with 200 μM and 400 μM A-769662, respectively, for 24 hours. Cells were detached with 0.25% trypsin-EDTA. 20,000 cells were plated in quadruplicate into the wells of the xCELLigence microwell E-plate (Acea Biosciences) in drug-containing media (serum-free media for compound c and full-serum media for A-769662). The plate was placed in the RTCA DP analyzer maintained at 37°C and 5% CO_2_. Electrical impedance, which corresponds to the Cell Index, was recorded every 4 minutes for 55 sweeps.

### Cytotoxicity assay

SRB cytotoxicity assay was done as previously published [35] with the following modifications: cells were fixed with 50% (w/v) TCA and stained with 0.4% (w/v) SRB solution for 10 minutes.

## SUPPLEMENTARY MATERIALS FIGURES



## References

[1] Husemann Y, Geigl JB, Schubert F, Musiani P, Meyer M, Burghart E, Forni G, Eils R, Fehm T, Riethmuller G, Klein CA (2008). Systemic spread is an early step in breast cancer. Cancer cell.

[2] Cristofanilli M, Budd GT, Ellis MJ, Stopeck A, Matera J, Miller MC, Reuben JM, Doyle GV, Allard WJ, Terstappen LW, Hayes DF (2004). Circulating tumor cells, disease progression, and survival in metastatic breast cancer. The New England journal of medicine.

[3] Cristofanilli M, Hayes DF, Budd GT, Ellis MJ, Stopeck A, Reuben JM, Doyle GV, Matera J, Allard WJ, Miller MC, Fritsche HA, Hortobagyi GN, Terstappen LW (2005). Circulating tumor cells: a novel prognostic factor for newly diagnosed metastatic breast cancer. Journal of clinical oncology : official journal of the American Society of Clinical Oncology.

[4] Eckhardt BL, Francis PA, Parker BS, Anderson RL (2012). Strategies for the discovery and development of therapies for metastatic breast cancer. Nature reviews Drug discovery.

[5] Hardie DG, Salt IP, Hawley SA, Davies SP (1999). AMP-activated protein kinase: an ultrasensitive system for monitoring cellular energy charge. The Biochemical journal.

[6] Hardie DG, Ross FA, Hawley SA (2012). AMPK: a nutrient and energy sensor that maintains energy homeostasis. Nature reviews Molecular cell biology.

[7] Buzzai M, Jones RG, Amaravadi RK, Lum JJ, DeBerardinis RJ, Zhao F, Viollet B, Thompson CB (2007). Systemic treatment with the antidiabetic drug metformin selectively impairs p53-deficient tumor cell growth. Cancer research.

[8] Alimova IN, Liu B, Fan Z, Edgerton SM, Dillon T, Lind SE, Thor AD (2009). Metformin inhibits breast cancer cell growth, colony formation and induces cell cycle arrest *in vitro*. Cell cycle.

[9] Jones RG, Plas DR, Kubek S, Buzzai M, Mu J, Xu Y, Birnbaum MJ, Thompson CB (2005). AMP-activated protein kinase induces a p53-dependent metabolic checkpoint. Molecular cell.

[10] Hadad SM, Hardie DG, Appleyard V, Thompson AM (2014). Effects of metformin on breast cancer cell proliferation, the AMPK pathway and the cell cycle. Clinical & translational oncology : official publication of the Federation of Spanish Oncology Societies and of the National Cancer Institute of Mexico.

[11] Huang X, Wullschleger S, Shpiro N, McGuire VA, Sakamoto K, Woods YL, McBurnie W, Fleming S, Alessi DR (2008). Important role of the LKB1-AMPK pathway in suppressing tumorigenesis in PTEN-deficient mice. The Biochemical journal.

[12] Thompson AM (2014). Molecular Pathways: Preclinical Models and Clinical Trials with Metformin in Breast Cancer. Clinical cancer research : an official journal of the American Association for Cancer Research.

[13] Chen S, Zhu X, Lai X, Xiao T, Wen A, Zhang J (2014). Combined cancer therapy with non-conventional drugs: all roads lead to AMPK. Mini reviews in medicinal chemistry.

[14] Fogarty S, Hardie DG (2010). Development of protein kinase activators: AMPK as a target in metabolic disorders and cancer. Biochimica et biophysica acta.

[15] Hekimian K, Meisezahl S, Trompelt K, Rabenstein C, Pachmann K (2012). Epithelial cell dissemination and readhesion: analysis of factors contributing to metastasis formation in breast cancer. ISRN oncology.

[16] Labelle M, Hynes RO (2012). The initial hours of metastasis: the importance of cooperative host-tumor cell interactions during hematogenous dissemination. Cancer discovery.

[17] Korb T, Schluter K, Enns A, Spiegel HU, Senninger N, Nicolson GL, Haier J (2004). Integrity of actin fibers and microtubules influences metastatic tumor cell adhesion. Experimental cell research.

[18] Morris VL, MacDonald IC, Koop S, Schmidt EE, Chambers AF, Groom AC (1993). Early interactions of cancer cells with the microvasculature in mouse liver and muscle during hematogenous metastasis: videomicroscopic analysis. Clinical & experimental metastasis.

[19] Matrone MA, Whipple RA, Balzer EM, Martin SS (2010). Microtentacles tip the balance of cytoskeletal forces in circulating tumor cells. Cancer research.

[20] Matrone MA, Whipple RA, Thompson K, Cho EH, Vitolo MI, Balzer EM, Yoon JR, Ioffe OB, Tuttle KC, Tan M, Martin SS (2010). Metastatic breast tumors express increased tau, which promotes microtentacle formation and the reattachment of detached breast tumor cells. Oncogene.

[21] Craig DH, Owen CR, Conway WC, Walsh MF, Downey C, Basson MD (2008). Colchicine inhibits pressure-induced tumor cell implantation within surgical wounds and enhances tumor-free survival in mice. The Journal of clinical investigation.

[22] Whipple RA, Cheung AM, Martin SS (2007). Detyrosinated microtubule protrusions in suspended mammary epithelial cells promote reattachment. Experimental cell research.

[23] Balzer EM, Whipple RA, Cho EH, Matrone MA, Martin SS (2010). Antimitotic chemotherapeutics promote adhesive responses in detached and circulating tumor cells. Breast cancer research and treatment.

[24] Vitolo MI, Boggs AE, Whipple RA, Yoon JR, Thompson K, Matrone MA, Cho EH, Balzer EM, Martin SS (2013). Loss of PTEN induces microtentacles through PI3K-independent activation of cofilin. Oncogene.

[25] Boggs AE, Vitolo MI, Whipple RA, Charpentier MS, Goloubeva OG, Ioffe OB, Tuttle KC, Slovic J, Lu Y, Mills GB, Martin SS (2015). alpha-Tubulin Acetylation Elevated in Metastatic and Basal-like Breast Cancer Cells Promotes Microtentacle Formation, Adhesion, and Invasive Migration. Cancer research.

[26] Janke C, Bulinski JC (2011). Post-translational regulation of the microtubule cytoskeleton: mechanisms and functions. Nature reviews Molecular cell biology.

[27] Akella JS, Wloga D, Kim J, Starostina NG, Lyons-Abbott S, Morrissette NS, Dougan ST, Kipreos ET, Gaertig J (2010). MEC-17 is an alpha-tubulin acetyltransferase. Nature.

[28] Webster DR, Gundersen GG, Bulinski JC, Borisy GG (1987). Differential turnover of tyrosinated and detyrosinated microtubules. Proceedings of the National Academy of Sciences of the United States of America.

[29] Whipple RA, Matrone MA, Cho EH, Balzer EM, Vitolo MI, Yoon JR, Ioffe OB, Tuttle KC, Yang J, Martin SS (2010). Epithelial-to-mesenchymal transition promotes tubulin detyrosination and microtentacles that enhance endothelial engagement. Cancer research.

[30] Balzer EM, Whipple RA, Thompson K, Boggs AE, Slovic J, Cho EH, Matrone MA, Yoneda T, Mueller SC, Martin SS (2010). c-Src differentially regulates the functions of microtentacles and invadopodia. Oncogene.

[31] Kusano K, Abe H, Obinata T (1999). Detection of a sequence involved in actin-binding and phosphoinositide-binding in the N-terminal side of cofilin. Molecular and cellular biochemistry.

[32] Miranda L, Carpentier S, Platek A, Hussain N, Gueuning MA, Vertommen D, Ozkan Y, Sid B, Hue L, Courtoy PJ, Rider MH, Horman S (2010). AMP-activated protein kinase induces actin cytoskeleton reorganization in epithelial cells. Biochemical and biophysical research communications.

[33] Nakano A, Kato H, Watanabe T, Min KD, Yamazaki S, Asano Y, Seguchi O, Higo S, Shintani Y, Asanuma H, Asakura M, Minamino T, Kaibuchi K, Mochizuki N, Kitakaze M, Takashima S (2010). AMPK controls the speed of microtubule polymerization and directional cell migration through CLIP-170 phosphorylation. Nature cell biology.

[34] Pachmann K, Dengler R, Lobodasch K, Frohlich F, Kroll T, Rengsberger M, Schubert R, Pachmann U (2008). An increase in cell number at completion of therapy may develop as an indicator of early relapse: quantification of circulating epithelial tumor cells (CETC) for monitoring of adjuvant therapy in breast cancer. Journal of cancer research and clinical oncology.

[35] Vichai V, Kirtikara K (2006). Sulforhodamine B colorimetric assay for cytotoxicity screening. Nature protocols.

[36] Ziegler E, Hansen MT, Haase M, Emons G, Grundker C (2014). Generation of MCF-7 cells with aggressive metastatic potential *in vitro* and *in vivo*. Breast cancer research and treatment.

[37] Ocana OH, Corcoles R, Fabra A, Moreno-Bueno G, Acloque H, Vega S, Barrallo-Gimeno A, Cano A, Nieto MA (2012). Metastatic colonization requires the repression of the epithelial-mesenchymal transition inducer Prrx1. Cancer cell.

[38] Zhou G, Myers R, Li Y, Chen Y, Shen X, Fenyk-Melody J, Wu M, Ventre J, Doebber T, Fujii N, Musi N, Hirshman MF, Goodyear LJ, Moller DE (2001). Role of AMP-activated protein kinase in mechanism of metformin action. The Journal of clinical investigation.

[39] Jin J, Mullen TD, Hou Q, Bielawski J, Bielawska A, Zhang X, Obeid LM, Hannun YA, Hsu YT (2009). AMPK inhibitor Compound C stimulates ceramide production and promotes Bax redistribution and apoptosis in MCF7 breast carcinoma cells. Journal of lipid research.

[40] Sanders MJ, Ali ZS, Hegarty BD, Heath R, Snowden MA, Carling D (2007). Defining the mechanism of activation of AMP-activated protein kinase by the small molecule A-769662, a member of the thienopyridone family. The Journal of biological chemistry.

[41] Goransson O, McBride A, Hawley SA, Ross FA, Shpiro N, Foretz M, Viollet B, Hardie DG, Sakamoto K (2007). Mechanism of action of A-769662, a valuable tool for activation of AMP-activated protein kinase. The Journal of biological chemistry.

[42] Zhuang Y, Miskimins WK (2008). Cell cycle arrest in Metformin treated breast cancer cells involves activation of AMPK, downregulation of cyclin D1, and requires p27Kip1 or p21Cip1. Journal of molecular signaling.

[43] Luo Z, Zang M, Guo W (2010). AMPK as a metabolic tumor suppressor: control of metabolism and cell growth. Future oncology.

[44] Mialhe A, Lafanechere L, Treilleux I, Peloux N, Dumontet C, Bremond A, Panh MH, Payan R, Wehland J, Margolis RL, Job D (2001). Tubulin detyrosination is a frequent occurrence in breast cancers of poor prognosis. Cancer research.

[45] Saba NF, Magliocca KR, Kim S, Muller S, Chen Z, Owonikoko TK, Sarlis NJ, Eggers C, Phelan V, Grist WJ, Chen AY, Ramalingam SS, Chen ZG, Beitler JJ, Shin DM, Khuri FR (2014). Acetylated tubulin (AT) as a prognostic marker in squamous cell carcinoma of the head and neck. Head Neck Pathol.

[46] Mackeh R, Lorin S, Ratier A, Mejdoubi-Charef N, Baillet A, Bruneel A, Hamai A, Codogno P, Pous C, Perdiz D (2014). Reactive oxygen species, AMP-activated protein kinase, and the transcription cofactor p300 regulate alpha-tubulin acetyltransferase-1 (alphaTAT-1/MEC-17)-dependent microtubule hyperacetylation during cell stress. The Journal of biological chemistry.

[47] Song Y, Brady ST (2015). Post-translational modifications of tubulin: pathways to functional diversity of microtubules. Trends in cell biology.

[48] Fassett JT, Hu X, Xu X, Lu Z, Zhang P, Chen Y, Bache RJ (2013). AMPK attenuates microtubule proliferation in cardiac hypertrophy. American journal of physiology Heart and circulatory physiology.

[49] Caino MC, Chae YC, Vaira V, Ferrero S, Nosotti M, Martin NM, Weeraratna A, O'Connell M, Jernigan D, Fatatis A, Languino LR, Bosari S, Altieri DC (2013). Metabolic stress regulates cytoskeletal dynamics and metastasis of cancer cells. The Journal of clinical investigation.

[50] Yamaguchi H, Lorenz M, Kempiak S, Sarmiento C, Coniglio S, Symons M, Segall J, Eddy R, Miki H, Takenawa T, Condeelis J (2005). Molecular mechanisms of invadopodium formation: the role of the N-WASP-Arp2/3 complex pathway and cofilin. The Journal of cell biology.

[51] Ghosh M, Song X, Mouneimne G, Sidani M, Lawrence DS, Condeelis JS (2004). Cofilin promotes actin polymerization and defines the direction of cell motility. Science.

[52] Sidani M, Wessels D, Mouneimne G, Ghosh M, Goswami S, Sarmiento C, Wang W, Kuhl S, El-Sibai M, Backer JM, Eddy R, Soll D, Condeelis J (2007). Cofilin determines the migration behavior and turning frequency of metastatic cancer cells. The Journal of cell biology.

[53] dos Remedios CG, Chhabra D, Kekic M, Dedova IV, Tsubakihara M, Berry DA, Nosworthy NJ (2003). Actin binding proteins: regulation of cytoskeletal microfilaments. Physiol Rev.

[54] Liang J, Mills GB (2013). AMPK: a contextual oncogene or tumor suppressor?. Cancer research.

[55] Laderoute KR, Amin K, Calaoagan JM, Knapp M, Le T, Orduna J, Foretz M, Viollet B (2006). 5′-AMP-activated protein kinase (AMPK) is induced by low-oxygen and glucose deprivation conditions found in solid-tumor microenvironments. Molecular and cellular biology.

[56] Kumar SH, Rangarajan A (2009). Simian virus 40 small T antigen activates AMPK and triggers autophagy to protect cancer cells from nutrient deprivation. Journal of virology.

[57] Jeon SM, Chandel NS, Hay N (2012). AMPK regulates NADPH homeostasis to promote tumour cell survival during energy stress. Nature.

[58] Shackelford DB, Shaw RJ (2009). The LKB1-AMPK pathway: metabolism and growth control in tumour suppression. Nature reviews Cancer.

[59] Faubert B, Boily G, Izreig S, Griss T, Samborska B, Dong Z, Dupuy F, Chambers C, Fuerth BJ, Viollet B, Mamer OA, Avizonis D, DeBerardinis RJ, Siegel PM, Jones RG (2013). AMPK is a negative regulator of the Warburg effect and suppresses tumor growth *in vivo*. Cell metabolism.

[60] Grassian AR, Coloff JL, Brugge JS (2011). Extracellular matrix regulation of metabolism and implications for tumorigenesis. Cold Spring Harbor symposia on quantitative biology.

[61] Ng TL, Leprivier G, Robertson MD, Chow C, Martin MJ, Laderoute KR, Davicioni E, Triche TJ, Sorensen PH (2012). The AMPK stress response pathway mediates anoikis resistance through inhibition of mTOR and suppression of protein synthesis. Cell death and differentiation.

[62] Wong CW, Lee A, Shientag L, Yu J, Dong Y, Kao G, Al-Mehdi AB, Bernhard EJ, Muschel RJ (2001). Apoptosis: an early event in metastatic inefficiency. Cancer research.

[63] Simpson CD, Anyiwe K, Schimmer AD (2008). Anoikis resistance and tumor metastasis. Cancer letters.

[64] Aceto N, Bardia A, Miyamoto DT, Donaldson MC, Wittner BS, Spencer JA, Yu M, Pely A, Engstrom A, Zhu H, Brannigan BW, Kapur R, Stott SL, Shioda T, Ramaswamy S, Ting DT (2014). Circulating tumor cell clusters are oligoclonal precursors of breast cancer metastasis. Cell.

