# Supplementary material for: Pharmacologic regulation of AMPK in breast cancer affects cytoskeletal properties involved with microtentacle formation and re-attachment
Source: Oncotarget. 2015 Sep 23;6(34):36292–307. doi: 10.18632/oncotarget.5345 (PMC4742178; doi:10.18632/oncotarget.5345)
Supplement: Supplementary file 1 [file oncotarget-06-36292-s001.pdf]

SUPPLEMENTARY FIGURES

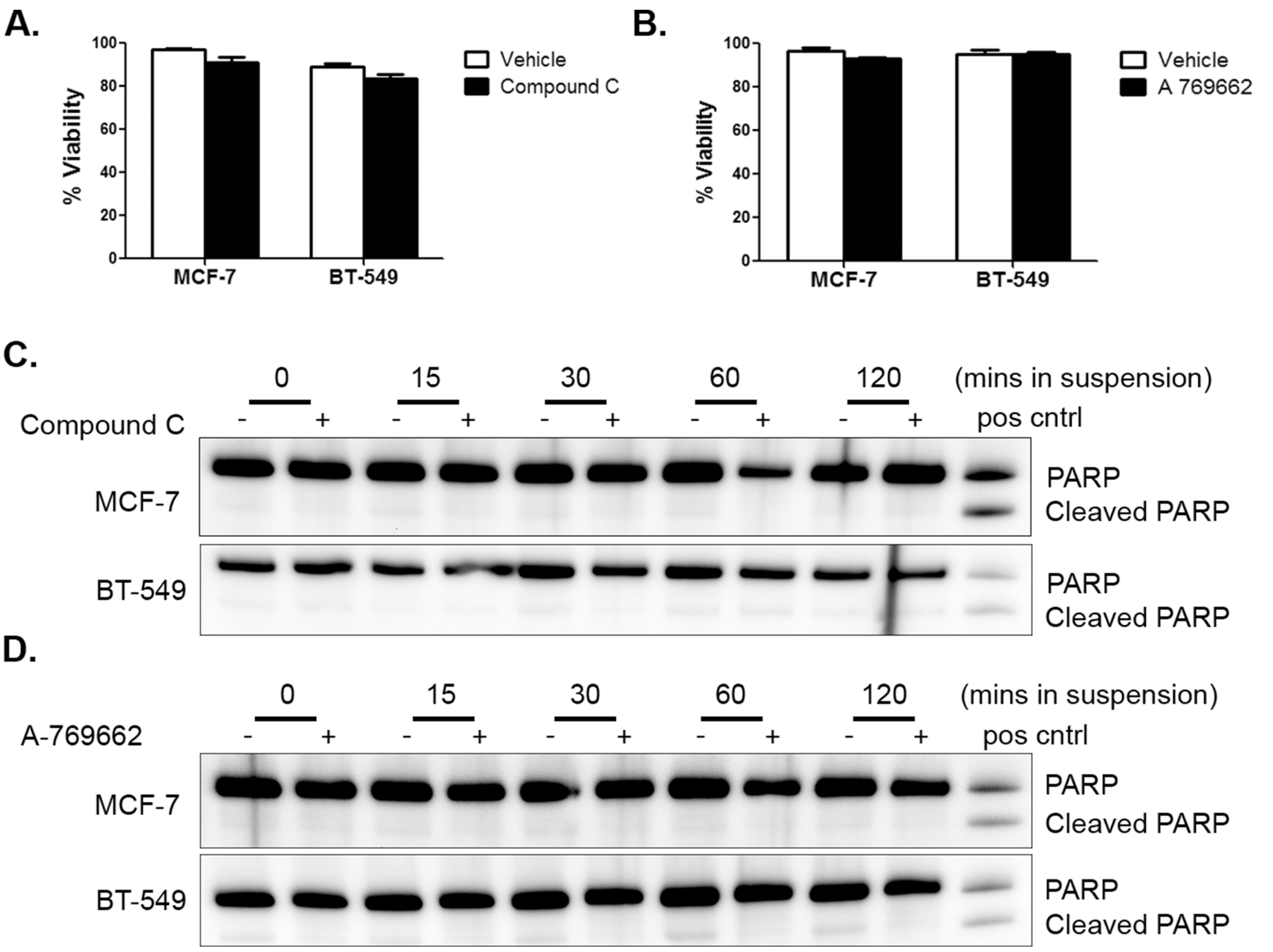

Supplementary Figure S1: AMPK does not induce cell death at early time points or in suspension. A. Trypan blue exclusion was assessed on MCF-7 and BT-549 cells after treatment with 20 µM compound c for 4 and 2 hours, respectively, in serum-free media. **B.** Trypan blue exclusion was assessed on MCF-7 and BT-549 cells after treatment with 200 µM and 400 µM A-769662, respectively, for 24 hours. **C.** MCF-7 and BT-549 cells were treated with 20 µM compound c in serum-free media for 4 and 2 hours, respectively, and then suspended. Cells were harvested immediately after suspension (0 min) and then harvested at 15, 30, 60, and 120 minutes. Western blot analysis was done for PARP cleavage. Positive control was BT-549 cells treated with 0.5 µM staurosporine for 5 hours. **D.** MCF-7 and BT-549 cells were treated with 200 µM and 400 µM A-769662, respectively, for 24 hours and then suspended. Cells were harvested immediately after suspension (0 min) and then harvested at 15, 30, 60, and 120 minutes. Western blot analysis was done for PARP cleavage. Positive control was BT-549 cells treated with 0.5 µM staurosporine for 5 hours.

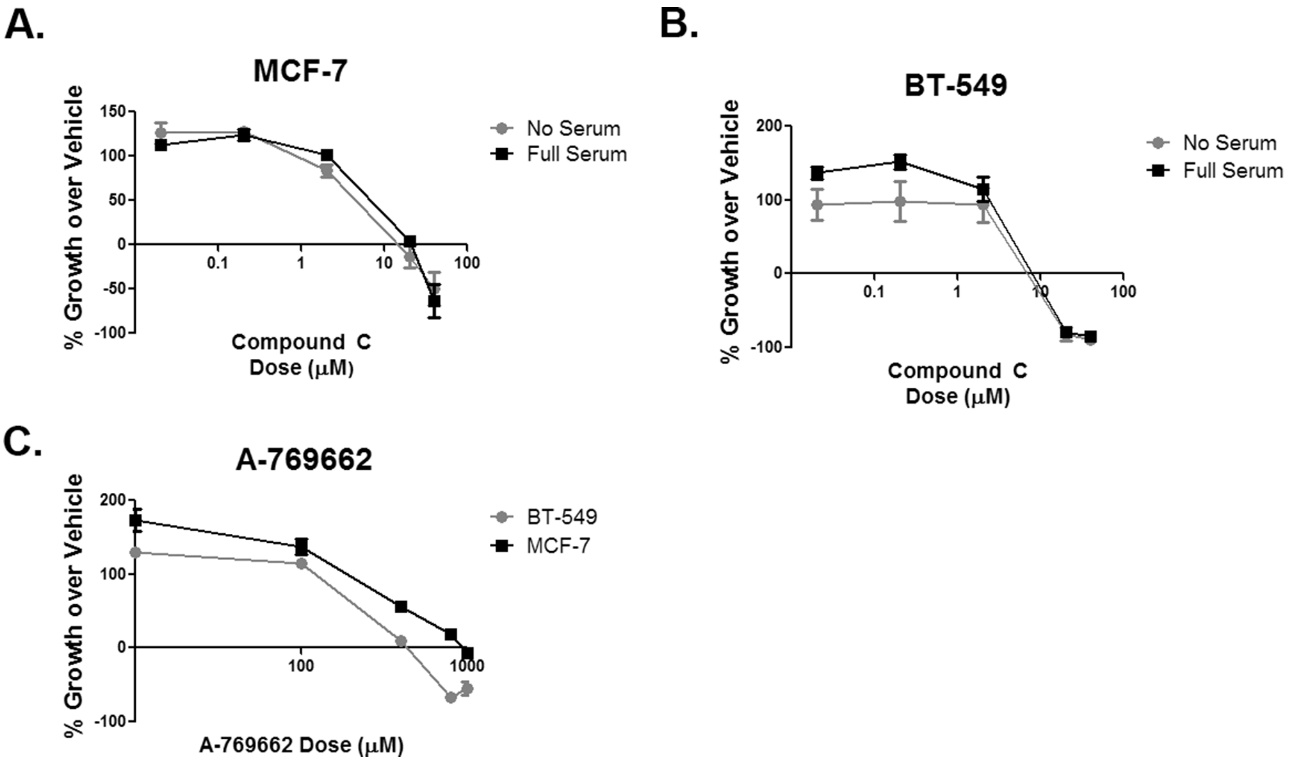

Supplementary Figure S2: Compound c and A-769662 inhibit cell growth. A & B. MCF-7 and BT-549 cells were treated with 20 nM–40 µM compound c for 48 hours in both full-serum and serum-free conditions. SRB assay was done and percent cell growth was determined by normalizing to vehicle control and day 0 untreated controls. Mean +/− SEM are shown from the combination of three independent experiments. **C.** MCF-7 and BT-549 cells were treated with 1 µM–1 mM A-769662 for 48 hours. SRB assay was done and percent cell growth was determined by normalizing to vehicle control and day 0 untreated controls. Mean +/− SEM are shown from the combination of three independent experiments.

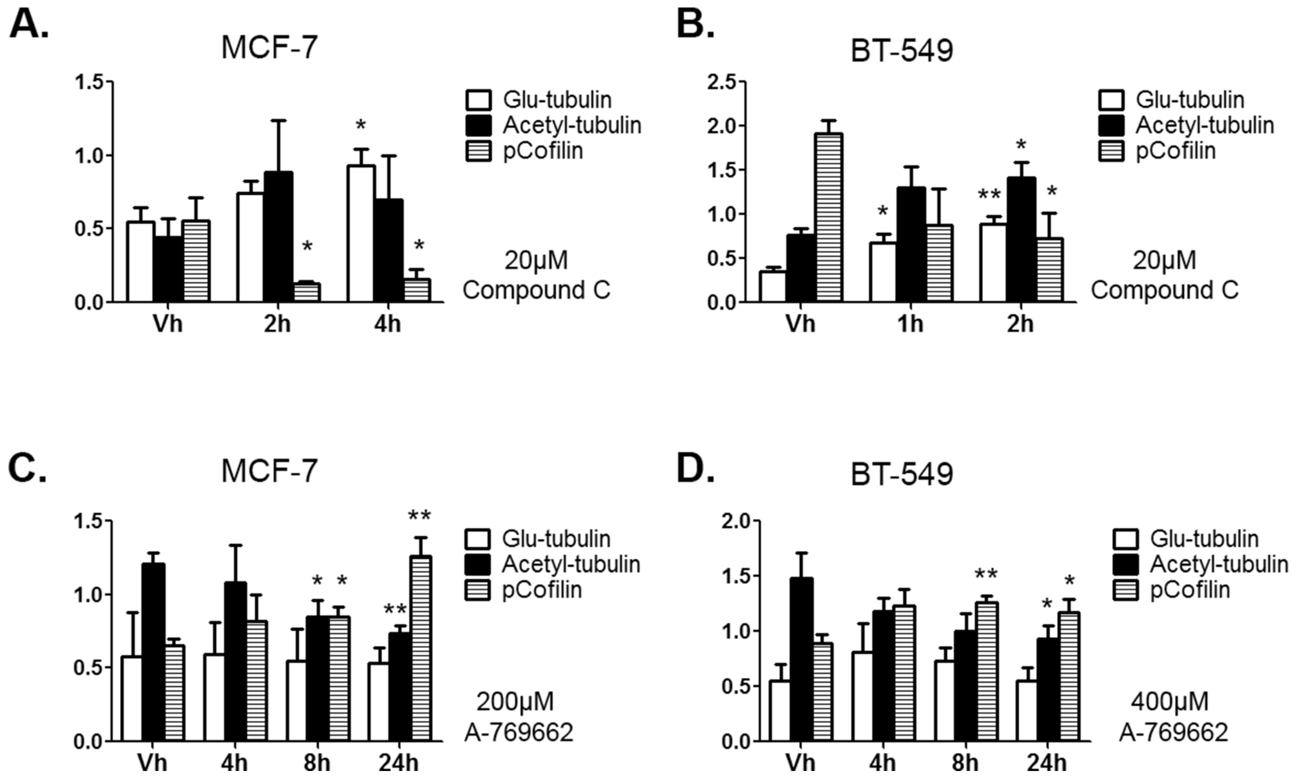

Supplementary Figure S3: AMPK changes microtubule stability and cofilin activation over time. A & B. Densitometry of MCF-7 and BT-549 cells treated with 20 µM compound c in serum-free media was done on three independent experiments (mean +/− SEM) to quantify levels of pCofilin, glu-tubulin, and acetylated tubulin over time with Western blot analysis. $*p < 0.05$ $**p < 0.005$ compared to vehicle. **C & D.** Densitometry of MCF-7 and BT-549 cells treated with 200 µM and 400 µM A-769662, respectively, was done on three independent experiments (mean +/− SEM) to quantify levels of pCofilin, glu-tubulin, and acetylated tubulin over time with Western blot analysis. $*p < 0.05$ $**p < 0.005$ compared to vehicle.

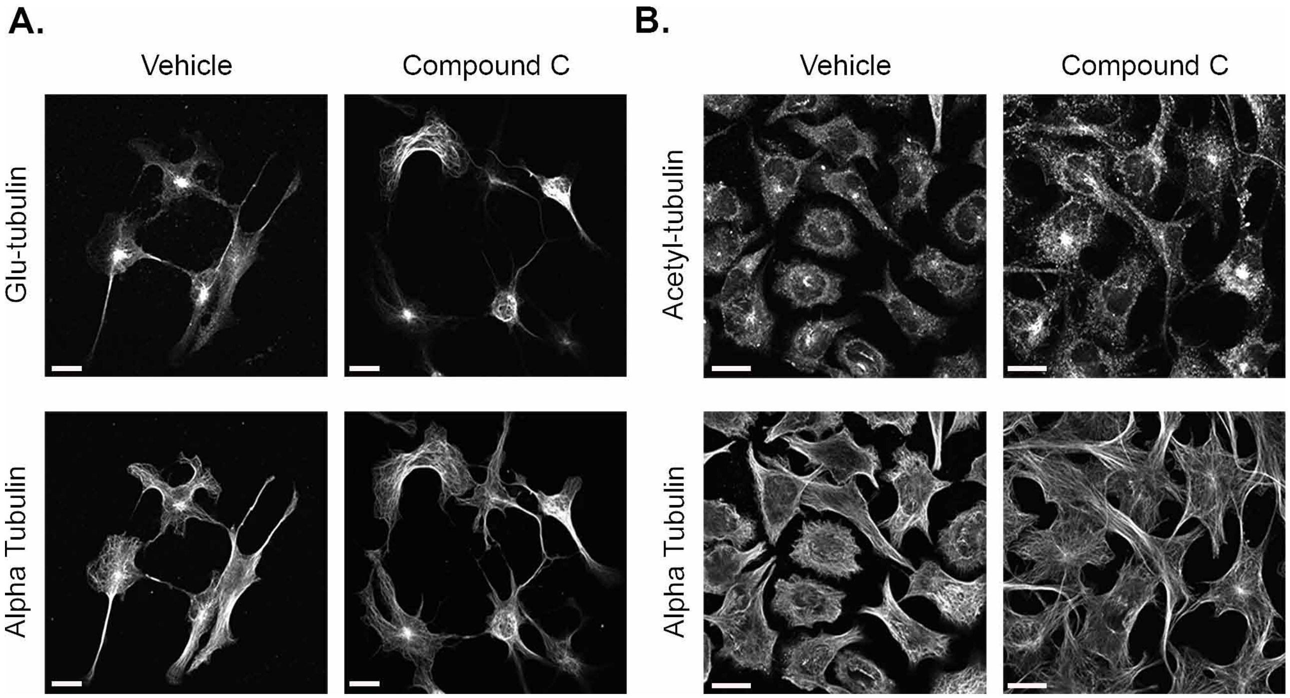

Supplementary Figure S4: Compound c increases glu-tubulin and acetylated tubulin levels in BT-549 cells. BT-549 cells treated with 20 µM compound c for 2 hours in serum-free media were fixed and stained with **A.** glu-tubulin and alpha tubulin. **B.** Acetylated tubulin and alpha tubulin. All images were taken at 60x magnification and are displayed as maximum z projections. Scale bar corresponds to 20 µm.

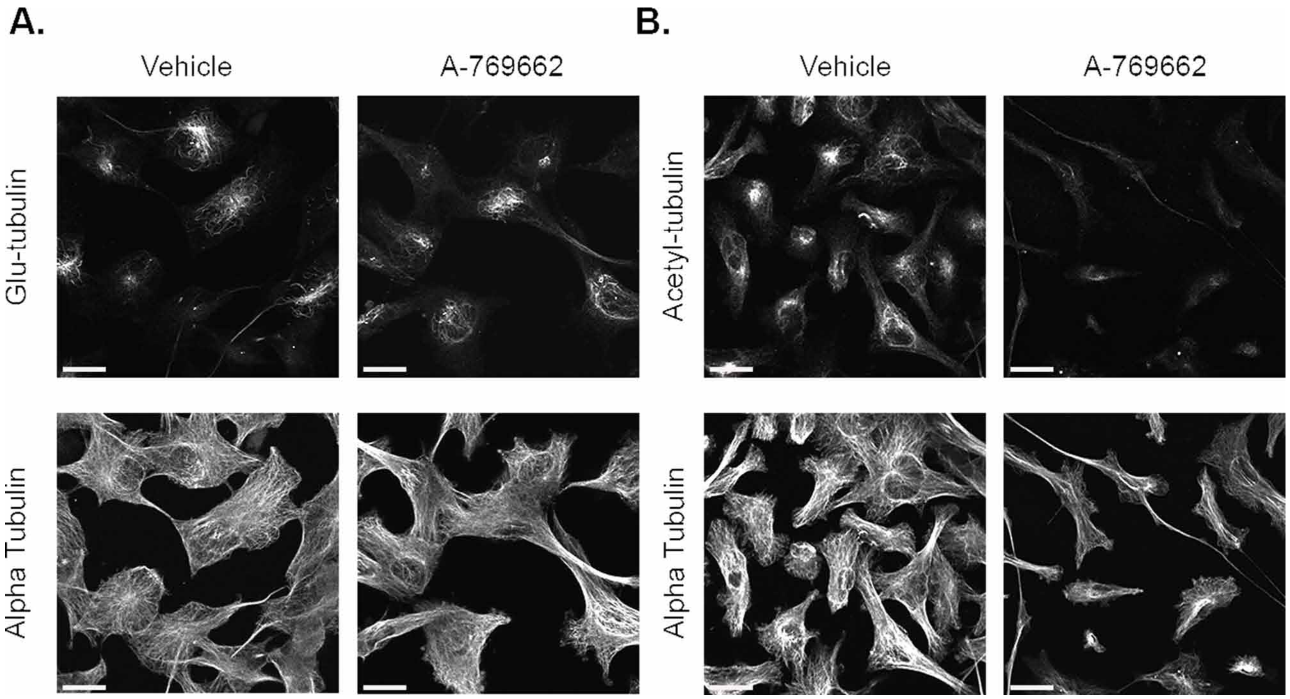

Supplementary Figure S5: A-769662 decreases acetylated tubulin in BT-549 cells. BT-549 cells treated with 400 µM A-769662 for 24 hours were fixed and stained with **A.** glu-tubulin and alpha tubulin. **B.** Acetylated tubulin and alpha tubulin. All images were taken at 60× magnification and are displayed as maximum z projections. Scale bar corresponds to 20 µm.
